# Supplementary material for: A Coarse-Grained MD Model for Disorder-To-Order Transitions in PolyQ Aggregation
Source: J Chem Theory Comput. 2025 Aug 1;21(19):9657–68. doi: 10.1021/acs.jctc.5c00384 (PMC12529903; doi:10.1021/acs.jctc.5c00384)
Supplement: Supplementary file 1 [file ct5c00384_si_001.pdf]

Supporting Information

A coarse-grained MD model for disorder-to-order transitions in polyQ aggregation

Maurice Dekker, Mark L. van der Klok, Erik Van der Giessen, Patrick R. Onck*

Zernike Institute for Advanced Materials, University of Groningen, 9747 AG, Groningen, The Netherlands
* Correspondence: p.r.onck@rug.nl

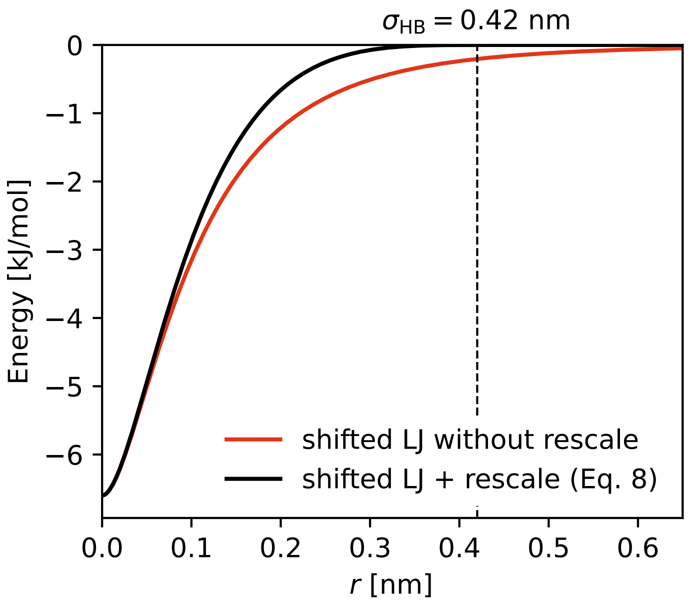

Fig. S1. Effect of the scale function on the hydrogen bonding potential. The scale function (Eq. 9) ensures that the potential is zero at $\sigma_{HB} = 0.42\,\text{nm}$.

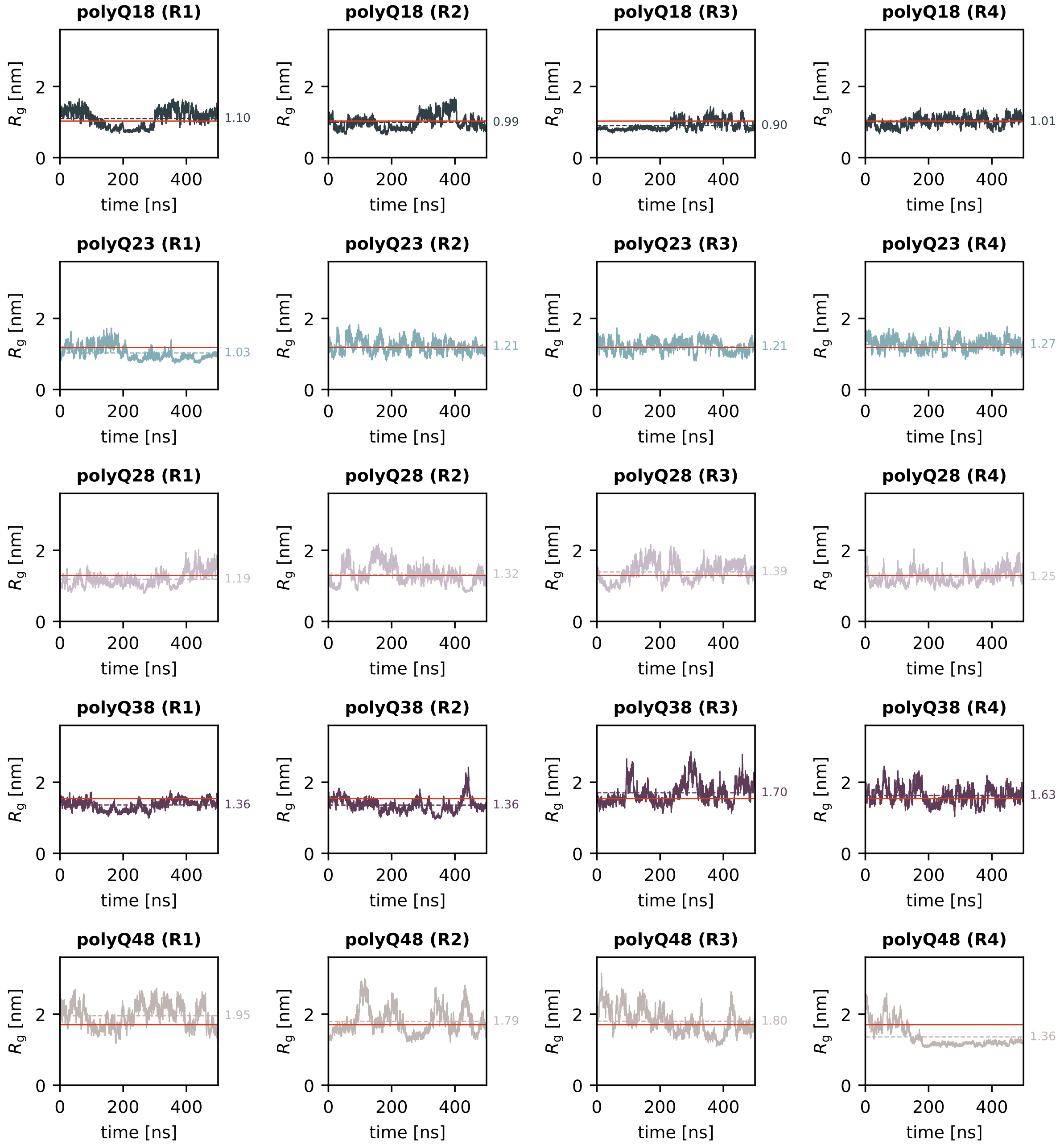

Fig. S2. Radius of gyration of monomeric polyglutamine (amber99SB-disp). The dashed lines indicate the average radius of gyration of each replica simulation, the solid red line marks the average across all eight replicas (both a99SB-disp and C36m) for that particular polyQ length; see black markers in Fig. 1f.

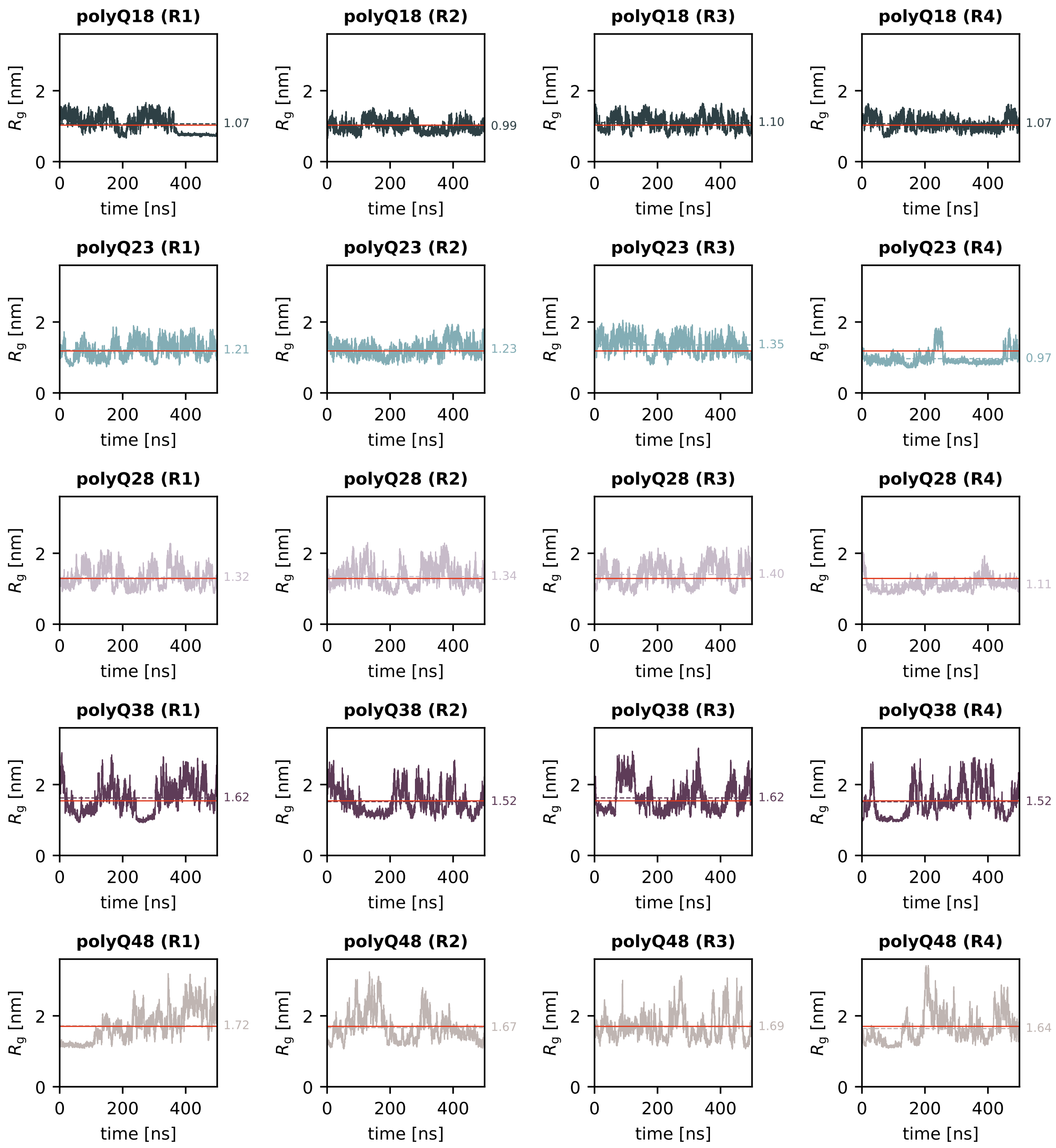

Fig. S3. Radius of gyration of monomeric polyglutamine (CHARMM36m). The dashed lines indicate the average radius of gyration of each replica simulation, the solid red line marks the average across all eight replicas (both a99SB-disp and C36m) for that particular polyQ length; see black markers in Fig. 1f.

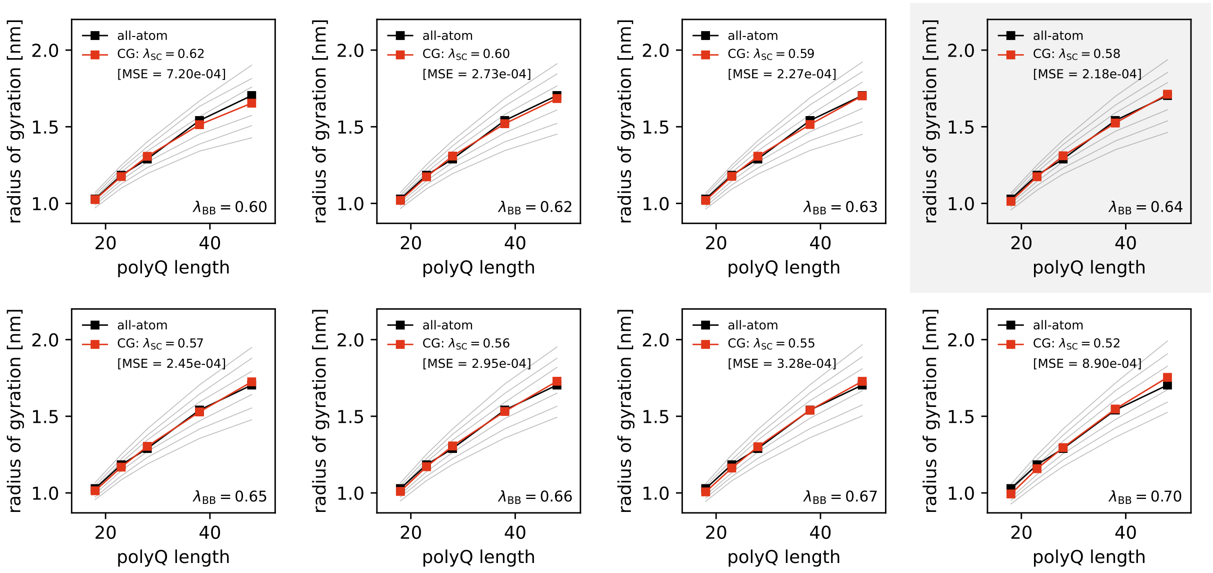

Fig. S4. Systematic calibration of backbone and side chain interaction strength in the 2BPA-Q model. For each backbone interaction λ_{BB} the R_g scaling is calculated for different side chain interactions (λ_{SC} increments of 0.01, gray lines) and the optimal side chain interaction strength is determined (red lines) by calculating the mean squared error from the all-atom measurements. There are multiple combinations of λ_{BB} and λ_{SC} that very accurately capture the R_g length dependence of polyQ. The optimal combination of $(\lambda_{BB}, \lambda_{SC})$ is where the MSE is the smallest, i.e. $(\lambda_{BB}, \lambda_{SC}) = (0.64, 0.58)$.

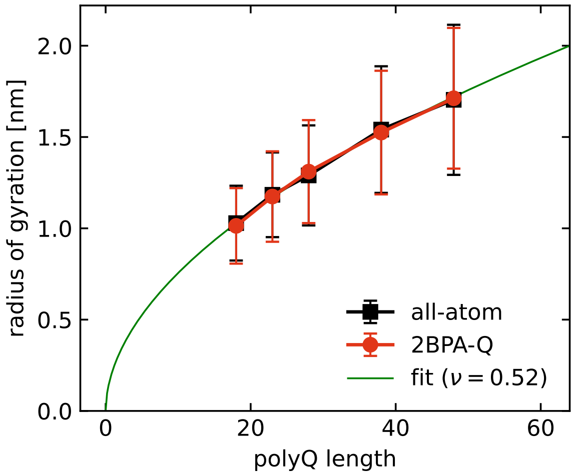

Fig. S5. Scaling of polyQ. The apparent scaling exponent is obtained from fitting $R_g = R_0 N^{\nu}$ to the gyration radii measured in the CG simulations, where N is the number of glutamine repeats and R_0 is the value corresponding to $N = 1$. The fitted scaling exponent is close to $\nu = 0.5$, which corresponds to an ideal random coil, where the protein chain has no significant interactions with itself and behaves like a non-interacting chain.

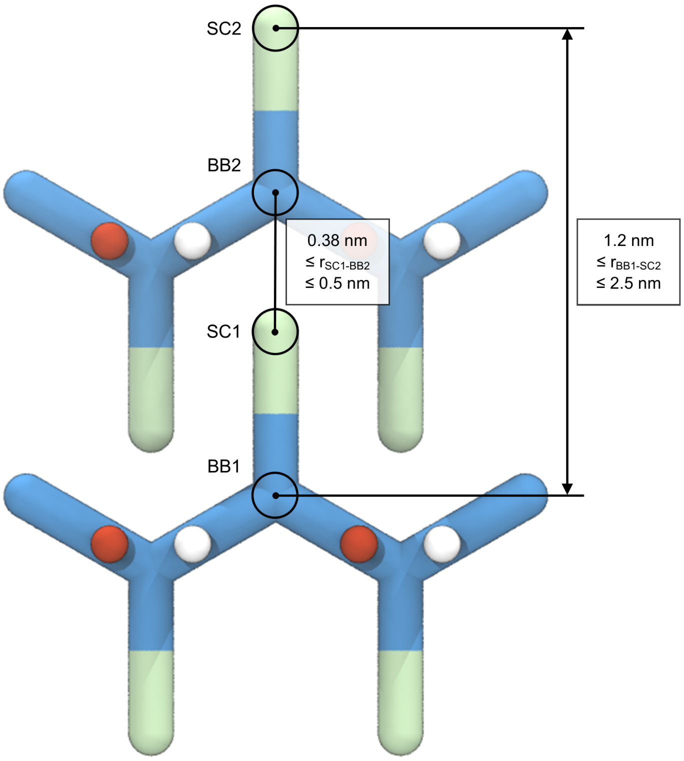

Fig. S6. Distance criteria for zipper conformation. Two distance conditions have to be satisfied for two residues to be in steric zipper conformation.

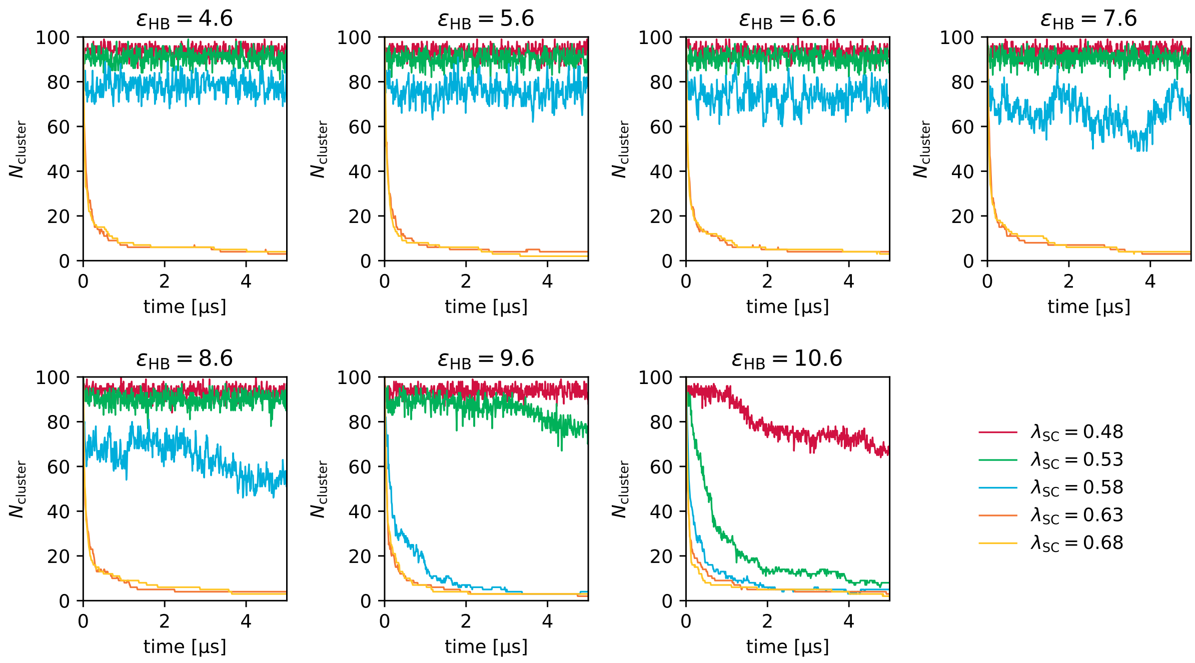

Fig. S7. Number of clusters as a function of time for all combinations of $(\lambda_{BB}, \lambda_{SC})$ in the phase diagram. For $\varepsilon_{HB} \leq 8.6\,$kJ/mol, the clustering behavior is primarily dictated by side chain interaction strength (λ_{SC}), with minimal influence from hydrogen bonding. At stronger hydrogen bonding energies ($\varepsilon_{HB} = 9.6$ and $10.6\,$kJ/mol), the λ_{SC} threshold for cluster formation decreases, indicating a synergistic effect between hydrogen bonding and nonspecific attractive interactions in driving cluster formation.

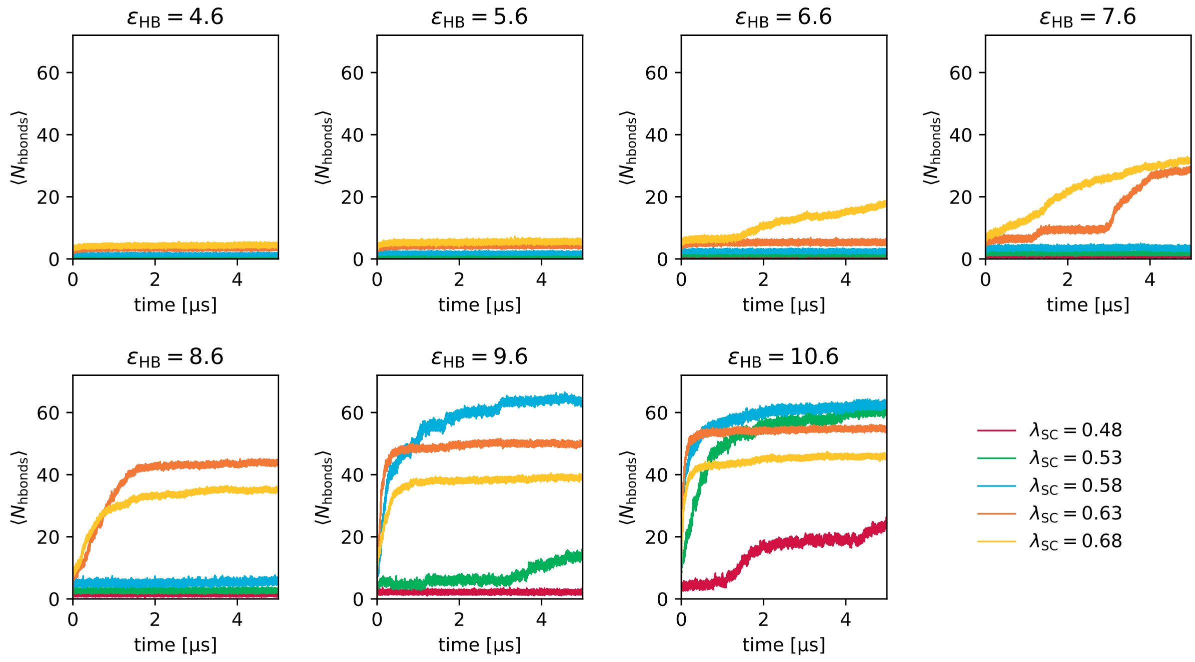

Fig. S8. Average number of hydrogen bonds per molecule as a function of time for all combinations of $(\lambda_{BB}, \lambda_{SC})$ in the phase diagram. At low hydrogen bonding energies ($\varepsilon_{HB} \leq 5.6\,$kJ/mol), no hydrogen bonds are formed. As ε_{HB} increases, hydrogen bonds form only after molecules have clustered due to side chain attraction. At stronger hydrogen bonding energies ($\varepsilon_{HB} = 9.6$ and $10.6\,$kJ/mol), different aggregation pathways are observed. For more attractive side chains ($\lambda_{SC} \geq 0.63$), clusters primarily form due to side chain interactions, while at lower interaction strength, both hydrogen bonding and nonspecific attractive interactions contribute to cluster formation, resulting in distinct aggregation behaviors and hydrogen bonding evolution curves.

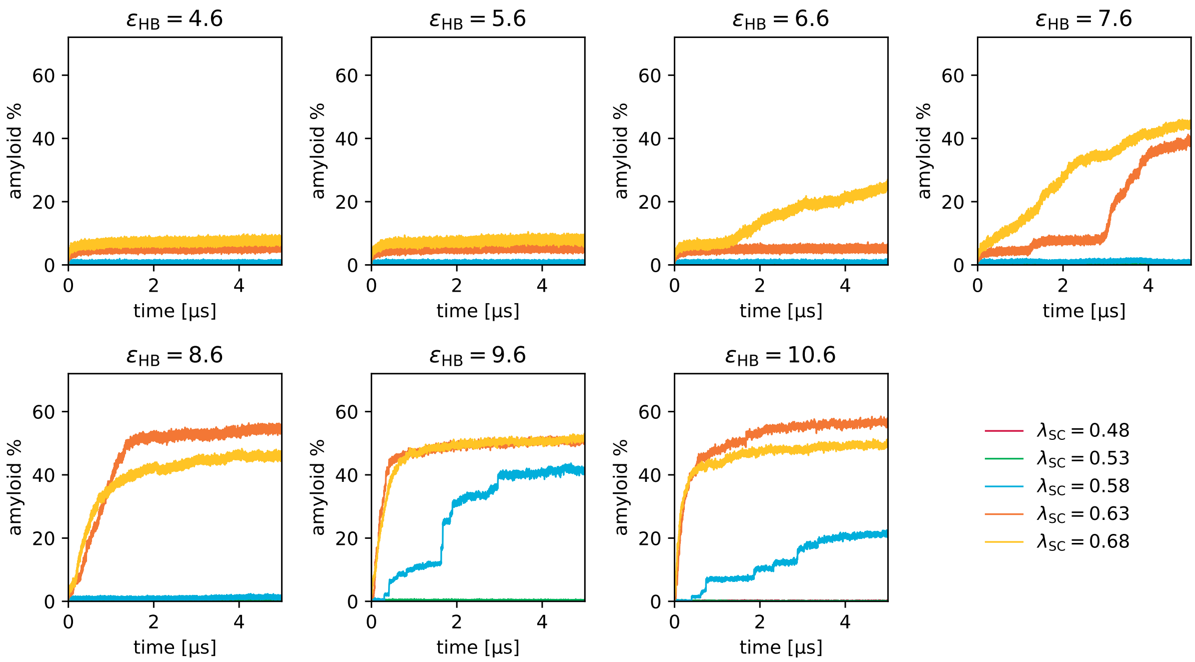

Fig. S9. Amyloid formation as a function of time for all combinations of ($\lambda_{BB}, \lambda_{SC}$) in the phase diagram. At low hydrogen bonding energies ($\varepsilon_{HB} \leq 5.6$ kJ/mol), no steric zippers are formed. As ε_{HB} increases, steric zippers form only after molecules have clustered due to side chain attraction, resulting in aggregation curves similar to those observed in the hydrogen bonding analysis. At stronger hydrogen bonding energies ($\varepsilon_{HB} = 9.6$ and 10.6 kJ/mol), steric zippers form only when the side chain interaction strength is sufficiently high ($\lambda_{SC} \geq 0.58$). For lower λ_{SC} values, β-sheets form, but steric zippers do not.

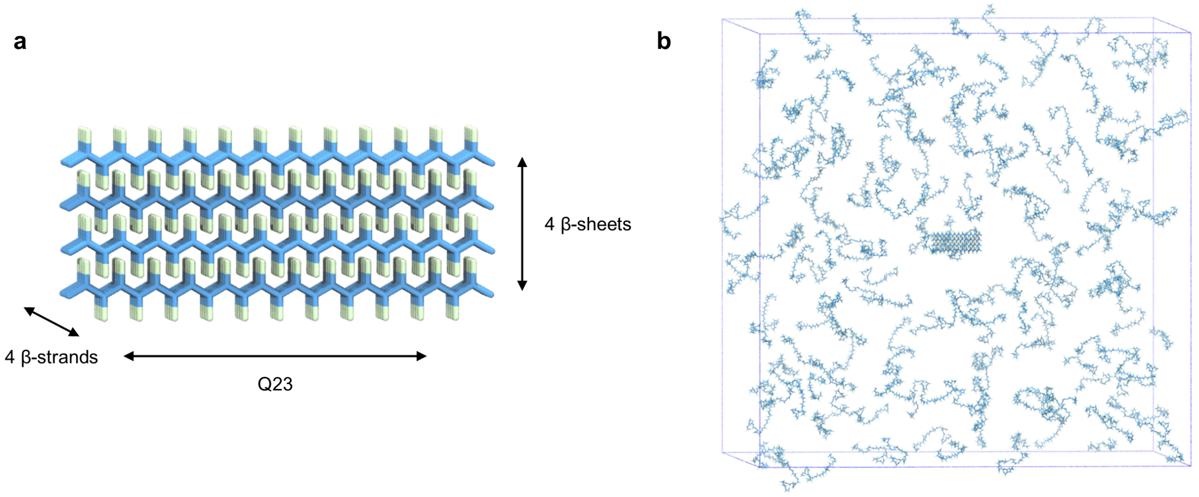

Fig. S10. Setup of seeded simulations. (**a**) The amyloid core used in the seeded simulations consists of a 4x4 antiparallel stack of Q23 monomers; hydrogen bonding beads are omitted for clarity. (**b**) The seed is placed in a simulation box containing 200 randomly distributed polyQ monomers at a molar concentration of 1.0 mM (box dimensions: 70 nm). The setup shown here corresponds to Q48 seeded simulations. Note that the amyloid core remains the same (4x4 antiparallel stack of Q23).

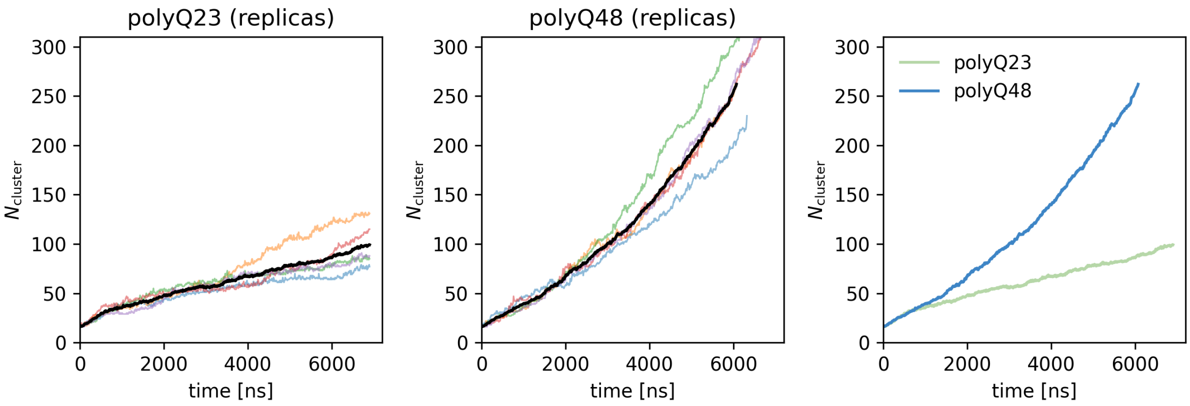

Fig. S11. Size of the largest cluster (number of molecules) for each of the replicas in the seeded aggregation simulations.

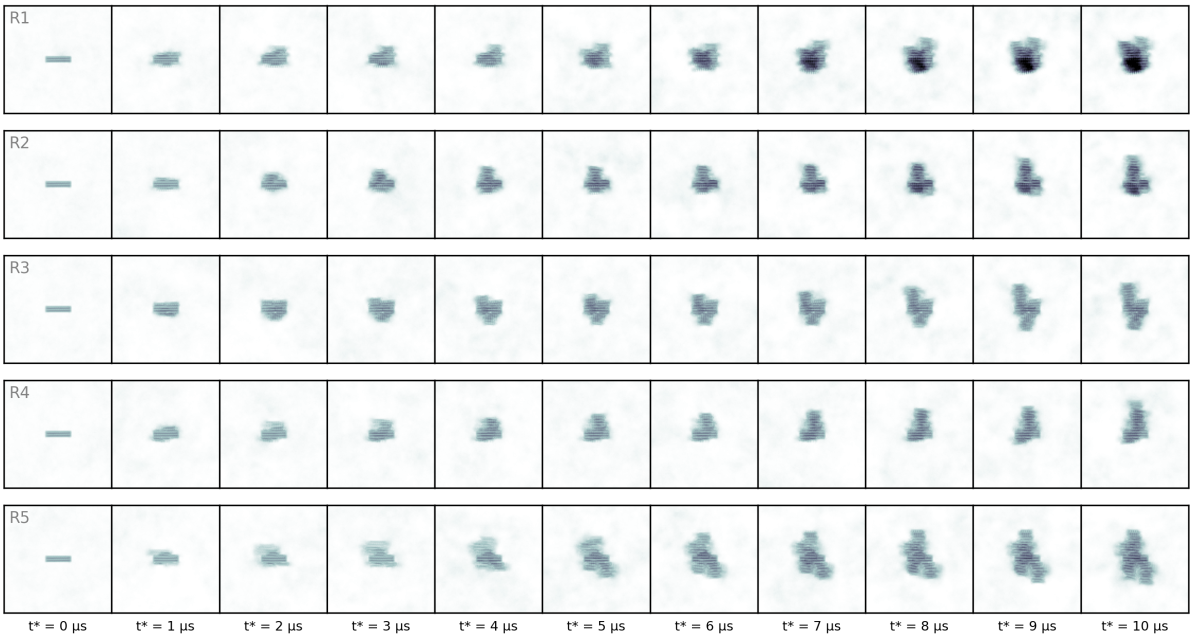

Fig. S12. Amyloid growth for five replicas of seeded simulations (polyQ23).

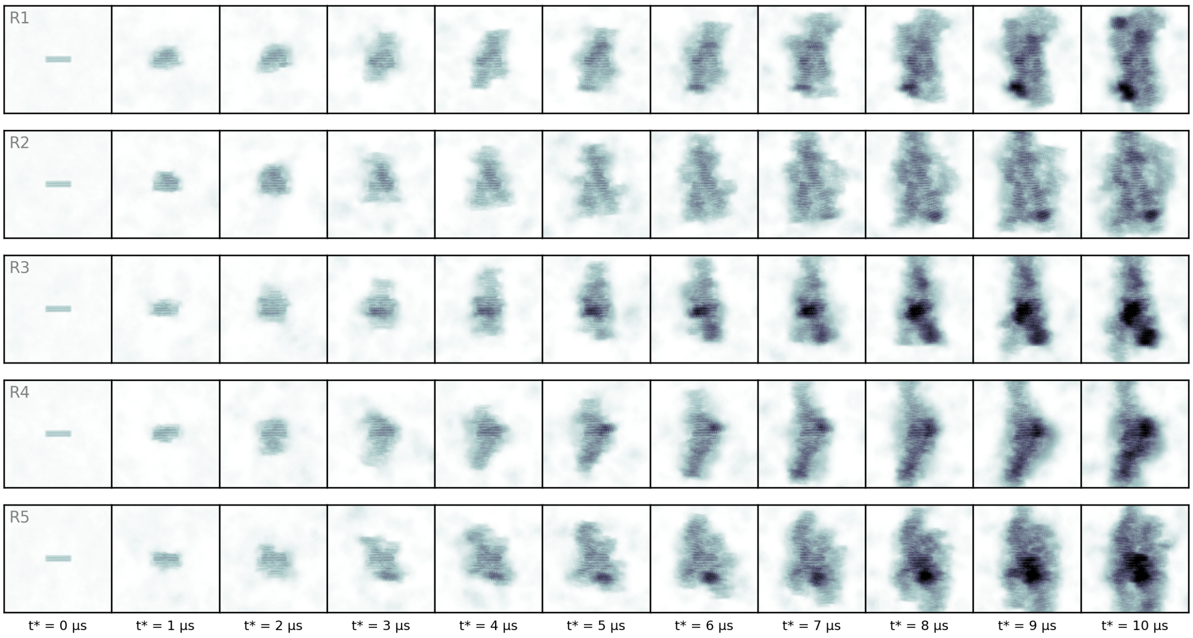

Fig. S13. Amyloid growth for five replicas of seeded simulations (polyQ48)

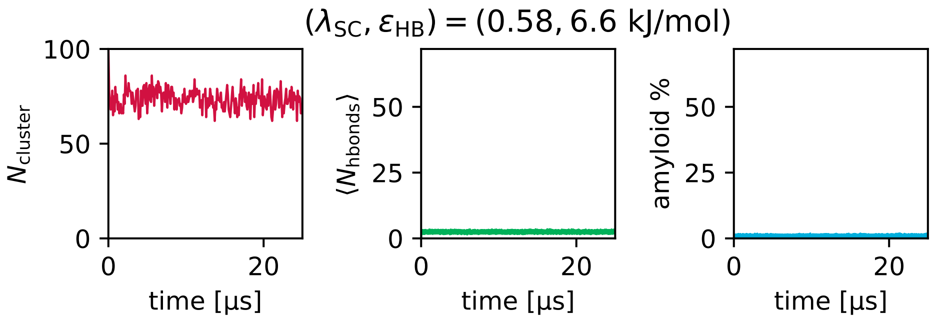

Fig. S14. Aggregation simulation analysis for a 25 μs **simulation using the default** $(\lambda_{\mathrm{SC}}, \varepsilon_{\mathrm{HB}})$ **values.**

Suppl. Movie S1. Animation of a simulation trajectory depicting amyloid growth through β-sheet elongation. The animation corresponds to the snapshots shown in Fig. 3a–c.

Suppl. Movie S2. Animation of a simulation trajectory depicting amyloid growth through the zipper mechanism. The animation corresponds to the snapshots shown in Fig. 3d–f.

Suppl. Movie S3. Two-dimensional animation of a simulation trajectory from a Q48 seeded simulation. The animation illustrates the first 5 microseconds of the seeded simulation (replica 3), which is also referenced in Fig. 4e.

References

1. Paulson, H.L.; Bonini, N.M.; Roth, K.A. Polyglutamine disease and neuronal cell death. *Proceedings of the National Academy of Sciences* **2000**, *97*, 12957–12958. doi:10.1073/pnas.210395797.
2. Zoghbi, H.Y.; Orr, H.T. Glutamine Repeats and Neurodegeneration. *Annual Review of Neuroscience* **2000**, *23*, 217–247. doi:10.1146/annurev.neuro.23.1.217.
3. Margolis, R.L.; Ross, C.A. Expansion explosion: new clues to the pathogenesis of repeat expansion neurodegenerative diseases. *Trends in Molecular Medicine* **2001**, *7*, 479–482. doi:10.1016/S1471-4914(01)02179-7.
4. Walker, F.O. Huntington's disease. *The Lancet* **2007**, *369*, 218–228. doi:10.1016/S0140-6736(07)60111-1.
5. Saudou, F.; Humbert, S. The Biology of Huntingtin. *Neuron* **2016**, *89*, 910–926. doi:10.1016/j.neuron.2016.02.003.
6. Gusella, J.F.; MacDonald, M.E. Molecular genetics: Unmasking polyglutamine triggers in neurodegenerative disease. *Nature Reviews Neuroscience* **2000**, *1*, 109–115. doi:10.1038/35039051.
7. Kuiper, E.F.E.; de Mattos, E.P.; Jardim, L.B.; Kampinga, H.H.; Bergink, S. Chaperones in Polyglutamine Aggregation: Beyond the Q-Stretch. *Frontiers in Neuroscience* **2017**, *11*, 145. doi:10.3389/fnins.2017.00145.
8. Chen, S.; Berthelier, V.; Yang, W.; Wetzel, R. Polyglutamine aggregation behavior in vitro supports a recruitment mechanism of cytotoxicity. *Journal of Molecular Biology* **2001**, *311*, 173–182. doi:10.1006/jmbi.2001.4850.
9. Chen, S.; Ferrone, F.A.; Wetzel, R. Huntington's disease age-of-onset linked to polyglutamine aggregation nucleation. *Proceedings of the National Academy of Sciences* **2002**, *99*, 11884–11889. doi:10.1073/pnas.182276099.
10. DiFiglia, M.; Sapp, E.; Chase, K.O.; Davies, S.W.; Bates, G.P.; Vonsattel, J.P.; Aronin, N. Aggregation of Huntingtin in Neuronal Intranuclear Inclusions and Dystrophic Neurites in Brain. *Science* **1997**, *277*, 1990–1993. doi:10.1126/science.277.5334.1990.
11. Kar, K.; Jayaraman, M.; Sahoo, B.; Kodali, R.; Wetzel, R. Critical nucleus size for disease-related polyglutamine aggregation is repeat-length dependent. *Nature Structural & Molecular Biology* **2011**, *18*, 328–336. doi:10.1038/nsmb.1992.
12. Kandola, T.; Venkatesan, S.; Zhang, J.; Lerbakken, B.T.; Von Schulze, A.; Blanck, J.F.; Wu, J.; Unruh, J.R.; Berry, P.; Lange, J.J.; et al. Pathologic polyglutamine aggregation begins with a self-poisoning polymer crystal. *eLife* **2023**, *12*. doi:10.7554/eLife.86939.
13. Peskett, T.R.; Rau, F.; O'Driscoll, J.; Patani, R.; Lowe, A.R.; Saibil, H.R. A Liquid to Solid Phase Transition Underlying Pathological Huntingtin Exon1 Aggregation. *Molecular Cell* **2018**, *70*, 588–601. doi:10.1016/j.molcel.2018.04.007.
14. Zhao, D.Y.; Bäuerlein, F.J.; Saha, I.; Hartl, F.U.; Baumeister, W.; Wilfling, F. Autophagy preferentially degrades non-fibrillar polyQ aggregates. *Molecular Cell* **2024**, *84*, 1980–1994. doi:10.1016/j.molcel.2024.04.018.
15. Legleiter, J.; Mitchell, E.; Lotz, G.P.; Sapp, E.; Ng, C.; DiFiglia, M.; Thompson, L.M.; Muchowski, P.J. Mutant Huntingtin Fragments Form Oligomers in a Polyglutamine Length-dependent Manner in Vitro and in Vivo. *Journal of Biological Chemistry* **2010**, *285*, 14777–14790. doi:10.1074/jbc.M109.093708.
16. Boatz, J.C.; Piretra, T.; Lasorsa, A.; Matlahov, I.; Conway, J.F.; van der Wel, P.C. Protofilament Structure and Supramolecular Polymorphism of Aggregated Mutant Huntingtin Exon 1. *Journal of Molecular Biology* **2020**, *432*, 4722–4744. doi:10.1016/j.jmb.2020.06.021.
17. Fodale, V.; Kegulian, N.C.; Verani, M.; Cariulo, C.; Azzollini, L.; Petricca, L.; Daldin, M.; Boggio, R.; Padova, A.; Kuhn, R.; et al. Polyglutamine- and Temperature-Dependent Conformational Rigidity in Mutant Huntingtin Revealed by Immunoassays and Circular Dichroism Spectroscopy. *PLoS ONE* **2014**, *9*, e112262. doi:10.1371/journal.pone.0112262.
18. Cui, X.; Liang, Q.; Liang, Y.; Lu, M.; Ding, Y.; Lu, B. TR-FRET Assays of Huntingtin Protein Fragments Reveal Temperature and PolyQ Length-Dependent Conformational Changes. *Scientific Reports* **2014**, *4*, 5601. doi:10.1038/srep05601.
19. Krobitsch, S.; Lindquist, S. Aggregation of huntingtin in yeast varies with the length of the polyglutamine expansion and the expression of chaperone proteins. *Proceedings of the National Academy of Sciences* **2000**, *97*, 1589–1594. doi:10.1073/pnas.97.4.1589.
20. Gillis, J.; Schipper-Krom, S.; Juenemann, K.; Gruber, A.; Coolen, S.; van den Nieuwendijk, R.; van Veen, H.; Overkleeft, H.; Goedhart, J.; Kampinga, H.H.; et al. The DNAJB6 and DNAJB8 Protein Chaperones Prevent Intracellular Aggregation of Polyglutamine Peptides. *Journal of Biological Chemistry* **2013**, *288*, 17225–17237. doi:10.1074/jbc.M112.421685.
21. Kakkar, V.; Månsson, C.; de Mattos, E.; Bergink, S.; van der Zwaag, M.; van Waarde, M.; Kloosterhuis, N.; Melki, R.; van Cruchten, R.; Al-Karadaghi, S.; et al. The S/T-Rich Motif in the DNAJB6 Chaperone Delays Polyglutamine Aggregation and the Onset of Disease in a Mouse Model. *Molecular Cell* **2016**, *62*, 272–283. doi:10.1016/j.molcel.2016.03.017.
22. Strodel, B. Amyloid aggregation simulations: challenges, advances and perspectives. *Current Opinion in Structural Biology* **2021**, *67*, 145–152. doi:10.1016/j.sbi.2020.10.019.
23. Ghavami, A.; Veenhoff, L.; Van der Giessen, E.; Onck, P.R. Probing the disordered domain of the nuclear pore complex through coarse-grained molecular dynamics simulations. *Biophysical Journal* **2014**, *107*, 1393–1402. doi:10.1016/j.bpj.2014.07.060.
24. Dignon, G.L.; Zheng, W.; Kim, Y.C.; Best, R.B.; Mittal, J. Sequence determinants of protein phase behavior from a coarse-grained model. *PLOS Computational Biology* **2018**, *14*, e1005941. doi:10.1371/journal.pcbi.1005941.
25. Joseph, J.A.; Reinhardt, A.; Aguirre, A.; Chew, P.Y.; Russell, K.O.; Espinosa, J.R.; Garaizar, A.; Collepardo-

Guevara, R. Physics-driven coarse-grained model for biomolecular phase separation with near-quantitative accuracy. *Nature Computational Science* **2021**, *1*, 732–743. doi:10.1038/s43588-021-00155-3.
26. Jafarinia, H.; Van der Giessen, E.; Onck, P.R. Phase separation of toxic dipeptide repeat proteins related to C9orf72 ALS/FTD. *Biophysical Journal* **2020**, *119*, 843–851. doi:10.1016/j.bpj.2020.07.005.
27. Das, S.; Lin, Y.H.; Vernon, R.M.; Forman-Kay, J.D.; Chan, H.S. Comparative roles of charge, <i>π</i> , and hydrophobic interactions in sequence-dependent phase separation of intrinsically disordered proteins. *Proceedings of the National Academy of Sciences* **2020**, *117*, 28795–28805. doi:10.1073/pnas.2008122117.
28. Tejedor, A.R.; Garaizar, A.; Ramírez, J.; Espinosa, J.R. 'RNA modulation of transport properties and stability in phase-separated condensates. *Biophysical Journal* **2021**, *120*, 5169–5186. doi:10.1016/j.bpj.2021.11.003.
29. Dekker, M.; Van der Giessen, E.; Onck, P.R. Phase separation of intrinsically disordered FG-Nups is driven by highly dynamic FG motifs. *Proceedings of the National Academy of Sciences* **2023**, *120*. doi:10.1073/pnas.2221804120.
30. Driver, M.D.; Postema, J.; Onck, P.R. The Effect of Dipeptide Repeat Proteins on FUS/TDP43-RNA Condensation in C9orf72 ALS/FTD. *The Journal of Physical Chemistry B* **2024**, *128*, 9405–9417. doi:10.1021/acs.jpcb.4c04663.
31. Szała-Mendyk, B.; Phan, T.M.; Mohanty, P.; Mittal, J. Challenges in studying the liquid-to-solid phase transitions of proteins using computer simulations. *Current Opinion in Chemical Biology* **2023**, *75*, 102333. doi:10.1016/j.cbpa.2023.102333.
32. Marchut, A.J.; Hall, C.K. Effects of chain length on the aggregation of model polyglutamine peptides: Molecular dynamics simulations. *Proteins: Structure, Function, and Bioinformatics* **2007**, *66*, 96–109. doi:10.1002/prot.21132.
33. Wang, Y.; Voth, G.A. Molecular Dynamics Simulations of Polyglutamine Aggregation Using Solvent-Free Multiscale Coarse-Grained Models. *The Journal of Physical Chemistry B* **2010**, *114*, 8735–8743. doi:10.1021/jp1007768.
34. Chen, M.; Tsai, M.; Zheng, W.; Wolynes, P.G. The Aggregation Free Energy Landscapes of Polyglutamine Repeats. *Journal of the American Chemical Society* **2016**, *138*, 15197–15203. doi:10.1021/jacs.6b08665.
35. Haaga, J.; Gunton, J.D.; Buckles, C.N.; Rickman, J.M. Early stage aggregation of a coarse-grained model of polyglutamine. *The Journal of Chemical Physics* **2018**, *148*, 045106. doi:10.1063/1.5010888.
36. Phan, T.M.; Schmit, J.D. Conformational entropy limits the transition from nucleation to elongation in amyloid aggregation. *Biophysical Journal* **2022**, *121*, 2931–2939. doi:10.1016/j.bpj.2022.06.031.
37. Nanajkar, N.; Sahoo, A.; Matysiak, S. Unraveling the Molecular Complexity of N-Terminus Huntingtin Oligomers: Insights into Polymorphic Structures. *The Journal of Physical Chemistry B* **2024**, *128*, 7761–7769. doi:10.1021/acs.jpcb.4c03274.
38. Szała-Mendyk, B.; Molski, A. Side Chain Geometry Determines the Fibrillation Propensity of a Minimal Two-Beads-per-Residue Peptide Model. *The Journal of Physical Chemistry B* **2022**, *126*, 5772–5780. doi:10.1021/acs.jpcb.2c03502.
39. Ghavami, A.; Van der Giessen, E.; Onck, P.R. Coarse-grained potentials for local interactions in unfolded proteins. *Journal of Chemical Theory and Computation* **2013**, *9*, 432–440. doi:10.1021/ct300684j.
40. Ananth, A.N.; Mishra, A.; Frey, S.; Dwarkasing, A.; Versloot, R.; van der Giessen, E.; Görlich, D.; Onck, P.; Dekker, C. Spatial structure of disordered proteins dictates conductance and selectivity in nuclear pore complex mimics. *eLife* **2018**, *7*, 1–24. doi:10.7554/eLife.31510.
41. Fragasso, A.; de Vries, H.W.; Andersson, J.; van der Sluis, E.O.; van der Giessen, E.; Dahlin, A.; Onck, P.R.; Dekker, C. A designer FG-Nup that reconstitutes the selective transport barrier of the nuclear pore complex. *Nature Communications* **2021**, *12*, 2010. doi:10.1038/s41467-021-22293-y.
42. Fragasso, A.; de Vries, H.W.; Andersson, J.; van der Sluis, E.O.; van der Giessen, E.; Onck, P.R.; Dekker, C. Transport receptor occupancy in nuclear pore complex mimics. *Nano Research* **2022**, *15*, 9689–9703. doi:10.1007/s12274-022-4647-1.
43. Hoop, C.L.; Lin, H.K.; Kar, K.; Magyarfalvi, G.; Lamley, J.M.; Boatz, J.C.; Mandal, A.; Lewandowski, J.R.; Wetzel, R.; van der Wel, P.C.A. Huntingtin exon 1 fibrils feature an interdigitated β-hairpin–based polyglutamine core. *Proceedings of the National Academy of Sciences* **2016**, *113*, 1546–1551. doi:10.1073/pnas.1521933113.
44. Sawaya, M.R.; Sambashivan, S.; Nelson, R.; Ivanova, M.I.; Sievers, S.A.; Apostol, M.I.; Thompson, M.J.; Balbirnie, M.; Wiltzius, J.J.W.; McFarlane, H.T.; et al. Atomic structures of amyloid cross-β spines reveal varied steric zippers. *Nature* **2007**, *447*, 453–457. doi:10.1038/nature05695.
45. Chen, J.Z.; Imamura, H. Universal model for α-helix and β-sheet structures in protein. *Physica A: Statistical Mechanics and its Applications* **2003**, *321*, 181–188. doi:10.1016/S0378-4371(02)01789-2.
46. Imamura, H.; Chen, J.Z.Y. Minimum model for the α-helix–β-hairpin transition in proteins. *Proteins: Structure, Function, and Bioinformatics* **2007**, *67*, 459–468. doi:10.1002/prot.21216.
47. Geddes, A.; Parker, K.; Atkins, E.; Beighton, E. "Cross-β" conformation in proteins. *Journal of Molecular Biology* **1968**, *32*, 343–358. doi:10.1016/0022-2836(68)90014-4.
48. Abraham, M.J.; Murtola, T.; Schulz, R.; Páll, S.; Smith, J.C.; Hess, B.; Lindahl, E. GROMACS: High performance molecular simulations through multi-level parallelism from laptops to supercomputers. *SoftwareX* **2015**, *1-2*, 19–25. doi:10.1016/j.softx.2015.06.001.
49. Sheu, S.Y.; Yang, D.Y.; Selzle, H.L.; Schlag, E.W. Energetics of hydrogen bonds in peptides. *Proceedings of the National Academy of Sciences* **2003**, *100*, 12683–12687. doi:10.1073/pnas.2133366100.
50. Kitchen, D.B.; Hirata, F.; Westbrook, J.D.; Levy, R.; Kofke, D.; Yarmush, M. Conserving energy during molecular dynamics simulations of water, proteins, and proteins in water. *Journal of Computational Chemistry* **1990**, *11*, 1169–1180. doi:10.1002/jcc.540111009.
51. Kmiecik, S.; Gront, D.; Kolinski, M.; Wieteska, L.; Dawid, A.E.; Kolinski, A. Coarse-Grained Protein Models and

Their Applications. *Chemical Reviews* **2016**, *116*, 7898–7936. doi:10.1021/acs.chemrev.6b00163.
52. Bagherpoor Helabad, M.; Matlahov, I.; Kumar, R.; Daldrop, J.O.; Jain, G.; Weingarth, M.; van der Wel, P.C.A.; Miettinen, M.S. Integrative determination of atomic structure of mutant huntingtin exon 1 fibrils implicated in Huntington disease. *Nature Communications* **2024**, *15*, 10793. doi:10.1038/s41467-024-55062-8.
53. Robustelli, P.; Piana, S.; Shaw, D.E. Developing a molecular dynamics force field for both folded and disordered protein states. *Proceedings of the National Academy of Sciences* **2018**, *115*, E4758–E4766. doi:10.1073/pnas.1800690115.
54. Huang, J.; Rauscher, S.; Nawrocki, G.; Ran, T.; Feig, M.; de Groot, B.L.; Grubmüller, H.; MacKerell, A.D. CHARMM36m: an improved force field for folded and intrinsically disordered proteins. *Nature Methods* **2017**, *14*, 71–73. doi:10.1038/nmeth.4067.
55. Bussi, G.; Donadio, D.; Parrinello, M. Canonical sampling through velocity rescaling. *The Journal of Chemical Physics* **2007**, *126*. doi:10.1063/1.2408420.
56. Parrinello, M.; Rahman, A. Polymorphic transitions in single crystals: A new molecular dynamics method. *Journal of Applied Physics* **1981**, *52*, 7182–7190. doi:10.1063/1.328693.
57. Darden, T.; York, D.; Pedersen, L. Particle mesh Ewald: An <i>N</i> log(<i>N</i>) method for Ewald sums in large systems. *The Journal of Chemical Physics* **1993**, *98*, 10089–10092. doi:10.1063/1.464397.
58. Hess, B.; Bekker, H.; Berendsen, H.J.C.; Fraaije, J.G.E.M. LINCS: A linear constraint solver for molecular simulations. *Journal of Computational Chemistry* **1997**, *18*, 1463–1472. doi:10.1002/(SICI)1096-987X(199709)18:12<1463::AID-JCC4>3.0.CO;2-H.
59. Gowers, R.; Linke, M.; Barnoud, J.; Reddy, T.; Melo, M.; Seyler, S.; Domański, J.; Dotson, D.; Buchoux, S.; Kenney, I.; et al. MDAnalysis: A Python Package for the Rapid Analysis of Molecular Dynamics Simulations. In Proceedings of the Proceedings of the 15th Python in Science Conference, 2016, pp. 98–105. doi:10.25080/Majora-629e541a-00e.
60. Humphrey, W.; Dalke, A.; Schulten, K. VMD: Visual molecular dynamics. *Journal of Molecular Graphics* **1996**, *14*, 33–38. doi:10.1016/0263-7855(96)00018-5.
61. Larsen, J.A.; Barclay, A.; Vettore, N.; Klausen, L.K.; Mangels, L.N.; Coden, A.; Schmit, J.D.; Lindorff-Larsen, K.; Buell, A.K. The mechanism of amyloid fibril growth from Φ-value analysis. *Nature Chemistry* **2025**, *17*, 403–411. doi:10.1038/s41557-024-01712-9.
62. Hutin, S.; Kumita, J.R.; Strotmann, V.I.; Dolata, A.; Ling, W.L.; Louafi, N.; Popov, A.; Milhiet, P.E.; Blackledge, M.; Nanao, M.H.; et al. Phase separation and molecular ordering of the prion-like domain of the Arabidopsis thermosensory protein EARLY FLOWERING 3. *Proceedings of the National Academy of Sciences* **2023**, *120*. doi:10.1073/pnas.2304714120.
63. Törnquist, M.; Michaels, T.C.T.; Sanagavarapu, K.; Yang, X.; Meisl, G.; Cohen, S.I.A.; Knowles, T.P.J.; Linse, S. Secondary nucleation in amyloid formation. *Chemical Communications* **2018**, *54*, 8667–8684. doi:10.1039/C8CC02204F.
64. Crick, S.L.; Ruff, K.M.; Garai, K.; Frieden, C.; Pappu, R.V. Unmasking the roles of N- and C-terminal flanking sequences from exon 1 of huntingtin as modulators of polyglutamine aggregation. *Proceedings of the National Academy of Sciences* **2013**, *110*, 20075–20080. doi:10.1073/pnas.1320626110.
65. Posey, A.E.; Ruff, K.M.; Harmon, T.S.; Crick, S.L.; Li, A.; Diamond, M.I.; Pappu, R.V. Profilin reduces aggregation and phase separation of huntingtin N-terminal fragments by preferentially binding to soluble monomers and oligomers. *Journal of Biological Chemistry* **2018**, *293*, 3734–3746. doi:10.1074/jbc.RA117.000357.
66. Heesink, G.; van den Oetelaar, M.C.M.; Semerdzhiev, S.A.; Ottmann, C.; Brunsveld, L.; Blum, C.; Claessens, M.M.A.E. 14-3-3τ as a Modulator of Early α-Synuclein Multimerization and Amyloid Formation. *ACS Chemical Neuroscience* **2024**, *15*, 1926–1936. doi:10.1021/acschemneuro.4c00100.
67. Hofmann, H.; Soranno, A.; Borgia, A.; Gast, K.; Nettels, D.; Schuler, B. Polymer scaling laws of unfolded and intrinsically disordered proteins quantified with single-molecule spectroscopy. *Proceedings of the National Academy of Sciences* **2012**, *109*, 16155–16160. doi:10.1073/pnas.1207719109.
68. Skeens, A.; Siriwardhana, C.; Massinople, S.E.; Wunder, M.M.; Ellis, Z.L.; Keith, K.M.; Girman, T.; Frey, S.L.; Legleiter, J. The polyglutamine domain is the primary driver of seeding in huntingtin aggregation. *PLOS ONE* **2024**, *19*, e0298323. doi:10.1371/journal.pone.0298323.
69. Phan, T.T.; Schmit, J.D. Thermodynamics of Huntingtin Aggregation. *Biophysical Journal* **2020**, *118*, 2989–2996. doi:10.1016/j.bpj.2020.05.013.
70. Buchanan, L.E.; Carr, J.K.; Fluitt, A.M.; Hoganson, A.J.; Moran, S.D.; de Pablo, J.J.; Skinner, J.L.; Zanni, M.T. Structural motif of polyglutamine amyloid fibrils discerned with mixed-isotope infrared spectroscopy. *Proceedings of the National Academy of Sciences* **2014**, *111*, 5796–5801. doi:10.1073/pnas.1401587111.
